# Photobiological modulation of hepatoma cell lines and hepatitis B subviral particles secretion in response to 650 nm low level laser treatment

**DOI:** 10.1186/s43046-023-00190-3

**Published:** 2023-10-23

**Authors:** Ghada M. Al-Toukhy, Reda A. Suef, Sarah Hassan, Mohamed M. S. Farag, Tarek A. El-Tayeb, Mohamed T. M. Mansour

**Affiliations:** 1 Department of Virology and Immunology, Children’s Cancer Hospital, Cairo, 57357 Egypt; 2 Department of Botany and Microbiology, Faculty of Science, Al-Azhar University, Cairo, 11884 Egypt; 3 Pathology and Electron Microscopy, Theodor Bilharz Research Institute, Giza, Egypt; 4 Biomedical Research Department, Armed Forces College of Medicine, Cairo, Egypt; 5 National Institute of Laser Enhanced Science (NILES), Cairo University, Cairo, Egypt; 6 Department of Virology and Immunology, National Cancer Institute, Cairo University, Cairo, Egypt; 7 Children Cancer Hospital, Cairo, 57357 Egypt

**Keywords:** Low-level laser therapy, Hepatitis B infection, Human hepatoma HepG2, HepG2.2.15 cell line, HBVsvps production, Photobiomodulation

## Abstract

**Background:**

Chronic hepatitis B virus (HBV) infection is a serious global health concern, with an increased incidence and risk of developing cirrhosis and hepatocellular carcinoma (HCC). Patients chronically infected with HBV are likely to experience chronic oxidative stress, leading to mitochondrial dysfunction. Photobiomodulation is induced by the absorption of low-level laser therapy (LLLT) with a red or infrared laser by cytochrome C oxidase enzyme, resulting in mitochondrial photoactivation. Although it is widely used in clinical practice, the use of LLL as adjuvant therapy for persistent HBV infection is uncommon. This study aimed to investigate the effect of LLLT dosage from 2 J/cm^2^ to 10 J/cm^2^ of red diode laser (650 nm) on both hepatoma cell lines (HepG2.2.15 [integrated HBV genome stable cell model] and non-integrated HepG2), with a subsequent impact on HBVsvp production.

**Methods:**

The present study evaluated the effects of different fluences of low-level laser therapy (LLLT) irradiation on various aspects of hepatoma cell behavior, including morphology, viability, ultrastructure, and its impact on HBVsvp synthesis.

**Results:**

In response to LLLT irradiation, we observed a considerable reduction in viability, proliferation, and HBVsvp production in both hepatoma cell lines HepG2.2.15 and HepG2. Ultrastructural modification of mitochondria and nuclear membranes: This effect was dose, cell type, and time-dependent.

**Conclusions:**

The use of LLLT may be a promising therapy for HCC and HBV patients by reducing cell proliferation, HBVsvp production, and altering mitochondrial and nuclear structure involved in cellular death inducers. Further research is required to explore its clinical application.

## Introduction

Chronic hepatitis B (CHB) is the leading cause of liver-associated morbidity and mortality worldwide. Approximately one-third of these patients develop liver failure, cirrhosis, and hepatocellular cancer (HCC) [1], if not adequately controlled and medically cared for, with approximately 650,000 people globally dying each year from various end-stage liver diseases caused by HBV infection [2]. During the acute phase of HBV infection, infected individuals' serum contains up to 100,000 folds of empty non-infectious spheres and filamentous subviral particles (SVPs) compared to the complete Dane virion (at 10^14^/mL) [3]. These SVPs play a role in HBV infection by acting as decoys for Dane particle-neutralizing antibodies, resulting in the immune tolerance required to maintain long-term chronic infection [4].

Human hepatocytes are known to be infected with HBV in a species- and tissue-specific manner [5]. Therefore, representative models of highly relevant cells and mice are necessary for the development and testing of novel antiviral medicines [6, 7]. The transformed hepatoma cell line HepG2.2.15, developed in 1987 by integrating an HBV genomic DNA fragment into HepG2 cells for the release of hepatitis B subviral particles (HBVsvp), has greatly aided our understanding of the HBV life cycle and viral-host interaction, as well as the evaluation of HBV antiviral agents [8]. Current anti-HBV medications are categorized into nucleoside/nucleotide analogs (NAs) and interferon-α (IFN-α), which have different mechanisms of action [9]. Although conventional therapy programs can reduce blood HBV levels below the detection limit of most clinical assays, the limited efficacy of HBV treatment, severe side effects, and the development of drug-resistant viral variants with long-term administration remain significant obstacles, necessitating the development and testing of new therapeutic approaches against HBV that achieve sustained suppression of HBV replication with alanine aminotransferase (ALT) normalization and liver histology improvement, leading to the reversal of fibrosis or cirrhosis, decrease in hepatic decompensation, and HCC [10].

Photobiomodulation therapy (PBM), also known as low-level laser therapy (LLLT), involves controlled utilization of red or near-infrared (NIR) laser light. This involves a wavelength of 600–1100 nm and an output power of 1–500 mW in a non-heating low energy density (0.04–50 J/cm^2^). The target tissue or cell monolayer is then exposed to the laser, which acts as a cellular photochemical stimulus when absorbed by a photoacceptor (such as the mitochondrial cytochrome c oxidase chromophore), promoting in vitro and in vivo biomodulatory effects that enhance adenosine triphosphate (ATP) production due to photochemical reactions in the cell [11]. This results in physiological changes and optogenetic modulation, which are considered the basis of many photobiological effects [12]. Furthermore, LLLT may modulate cellular redox systems by enhancing antioxidant enzymatic activity via a photochemical process that accelerates ROS elimination. Hence, pro-oxidant cells (such as HBV-infected hepatocytes) are more responsive to LLLT than are normal cells [13].

Several studies have reported that LLLT affects a variety of biological processes in cell cultures and animal models, including cell proliferation, metabolism, angiogenesis, apoptosis, and inflammation [14, 15]. Moreover, the beneficial effects of photobiomodulation are widely applied in different therapeutic conditions, such as improved muscle strength and functional performance [16], promoting skin wound healing [17], and dental therapy [18]. LLLT has also been used to control several aspects of viral infections. According to a study conducted on HIV-1 infected and uninfected TZM-bl cells, laser irradiation did not show an inhibitory effect in uninfected cells, but triggered cell damage in infected cells in a dose-dependent manner [19]. Furthermore, the effect of low-energy red laser (670 nm) on Herpes Simplex Type 1 (HSV-1) revealed that LLLT appears to be an effective treatment against (HSV-1) without observed side effects [20] and is proposed to act in the final stage of HSV-1 replication by limiting viral spread from cell to cell with modulation of the host’s immune response [21].

Despite the previous positive outcomes from clinical trials and laboratory studies, as shown above, LLLT has yet to be incorporated into mainstream medicine. Therefore, the objective of this study was to examine the PBM response of the hepatoma cell line HepG2.2.15 (an in vitro expression system for HBV) and non-integrated HepG2 cell lines to a low-level red diode laser under controlled irradiation and dose parameters that included (650 nm), CW mode, and dosages (2 J/cm^2^, 4 J/cm^2^, 8 J/cm^2^, and 10 J/cm^2^) at different time intervals of zero, 24, 48, and 96 h after laser treatment, and to correlate its effect on HBVsvps, which could be useful as an adjuvant antiviral photomedicine for the clinical management of CHB infection.

## Materials and methods

### Maintenance of cell lines

The human hepatocellular carcinoma (HCC) HepG2 cell line was purchased from American Type Culture Collection (ATCC), VACSERA, USA). HepG2 cells were propagated in a complete DMEM medium (Lonza, Swiss), which was supplemented with 100 U penicillin–streptomycin/mL (Sigma, USA), 100 μg L-glutamine/ml (Sigma, USA), and 10% heat inactivated fetal bovine serum (FBS) (Lonza, Belgium), incubated in 5% CO2 at 37 °C (Thermo, Germany), the cells up to 95% confluency. HepG2.2.15, genetically integrated with the S domain of the HBV genome, was used as a model to produce HBVsvps [6]. The hepatoma cell line HepG2.2.15 was grown in 10 ml of complete Williams’ E medium (Lonza, Switzerland) supplemented with 100 U penicillin–streptomycin (Sigma, USA), 100 μg L-glutamine/ml (Sigma, USA), 250 μL insulin (5 μg/ml) (Act rapid, Egypt), and 250 μL hydrocortisone (Sigma, USA). Next, 10% heat-inactivated (FBSfetal bovine serum; Lonza, Belgium) was incubated in 5% CO_2_ at 37 °C (Thermo Fisher Scientific). Both cell lines (HepG2 and HepG2.2.15) were incubated to assess the 90% confluency of cultivation. The cell monolayer was then washed twice, harvested by trypsinization using trypsin/EDTA (Lonza, Switzerland) at 37 °C [22], centrifuged at 1500 rpm for 5 min, the supernatant was discarded, and the cell pellet was resuspended in 1 ml fresh growth medium for counting.

### Trypan blue assay

The total number of cells per milliliter and the percentage of viable cells were determined using a hemocytometer and trypan blue vital stain. Viable cells (bright cells) were counted. In this assay, a 1:1 dilution was achieved by mixing an equal volume of 0.4% trypan blue reagent and cell suspension. The mixture was then transferred to a hemocytometer. Cells were counted and the viability percentage was calculated [23].

### Cell line cultivation setup

**Fig. 1:**
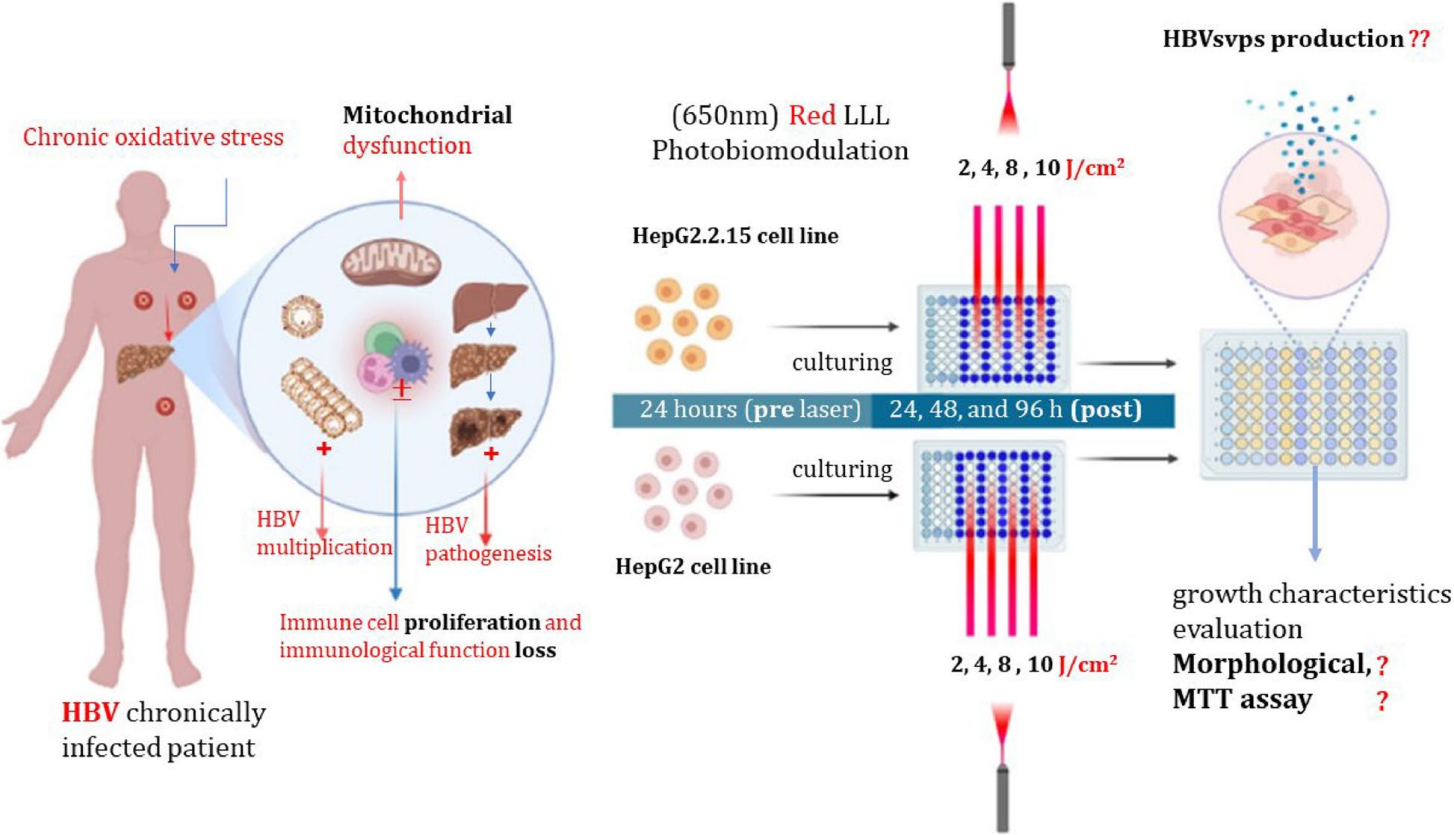
The experimental setup flowchart for the LLLI tests on both HepG2 and HepG2.2.15 cell lines

Before laser irradiation, each human hepatoma cell line (HepG2 and HepG2.2.15) was suspended in fresh 10% FBS complete culture medium. A volume of 100 μL of both cell suspensions (10 × 10^3^ cells density) was distributed into a 7-well culture plate (96-well/plate). The hepatoma cell lines HepG2 and HepG2.2.15 were cultured in five groups (A–E) in triplicate, based on the laser dose (fluence) and exposure time. Sterile water and blue ink were used to reduce media evaporation and prevent light transmission between groups. The cultured plates were incubated at 37 °C in 5% CO_2_ for 24 h before laser irradiation was performed (Fig. 1).

### Laser system setup and cell lines irradiation

The LLLT irradiation settings used in this study were established. In brief, the device employed was a continuous wave emission red diode laser device (Photonics, Egypt) with a wavelength of 650 nm, an average power output of 35 mW, and a spot size beam diameter of 0.2 cm^2^. The power density between the cover and cells was 0.035 W/cm at a distance of 0.4 cm. Prior to each plate irradiation, the laser beam was evaluated and measured using a power meter (SYNRAD, USA). Based on the laser dosages (Fluence) utilized, the cultured cells were divided into five groups: group A (not irradiated cells that serve as a control), group B irradiated with red diode laser 2 J/cm^2^, group C (4 J/cm^2^), group D (8 J/cm^2^), and group E (10 J/cm^2^) in ascending order according to the following equation

$$\mathrm{Fluence }\,(\mathrm{F}) = \frac{Power\,\left(P\right)W\,XTime\,\left(T\right)Second }{timArea\,(cm2) }=\mathrm{ J}/\mathrm{cm}^2$$

with the irradiation times at various fluences listed in (Table 1). All procedures were conducted in accordance with laser safety standards. Laser goggle (visible range) eyewear was used for eye protection, and laser irradiation was performed in full darkness. A laser power meter was used to determine the output power of the laser equipment.

**Table 1:** The parameters of the red diode laser employed in the present study for irradiation of HepG2 and HepG2.2.15 cell line (A–E) groups

Variable	Parameters
Wavelength	650 nm
Emission	Continuous wave
Power output	35 mW
Spot size diameter	0.2 cm
Exposure time	7.2 s, 14.4 s, 18.8 s, 36 s
Laser dosages	2 J/cm^2^, 4 J/cm^2^, 8 J/cm^2^, and 10 J/cm^2^

### Cell lines growth characteristics evaluation post-laser irradiation

#### Morphological examination

An inverted light microscope with a Leica digital camera (Germany) was used to examine changes in the hepatoma cell lines HepG2 and HepG2.2.15 morphology before laser irradiation and during each time incubation at zero time, 24 h, 48 h, and 96 h after laser irradiation.

#### MTT assay

The number of viable cells during the proliferation process was measured using an MTT assay. It involves the use of MTT reagent, which stands for 3-(4,5-dimethylthiazol-2-yl)-2,5-diphenyltetrazolium bromide (a yellowish powder that is soluble in water). When added to metabolically active living cells, MTT is reduced by mitochondrial enzymes (NAD(P)H-dependent oxidoreductase) to insoluble purple formazan crystals. The amount of formazan produced is proportional to the number of viable cells in the sample. To perform the MTT assay, MTT stock solution was prepared by dissolving 5 mg/ml in RPMI-1640 without phenol red (Lonza, Switzerland), filtered through a 0.2-m filter (Whatman, Germany), and storing at 2–8 °C for frequent use [24].

After laser irradiation, we measured cell viability using the MTT assay at several time points (zero-time, 24, 48, and 96 h): the supernatant from the variable time was collected. Then, 200 μl fresh medium (without phenol red), and 10 μL MTT stock solution were added to each well and incubated for 3 h. The cells were examined for punctate intracellular precipitates using an inverted microscope. When the purple precipitate was visible under the microscope, 200 μL of detergent reagent (DMSO) was added to all wells and left in the dark for 30 min at room temperature to dissolve the formazan salt [23–25]. The absorbance was measured in each well using an ELISA reader (Bio-Rad, USA) at a wavelength of 590 nm, with a reference wavelength of 650 nm. Optical density readings were averaged to create a single value. To assess the influence of low-level laser irradiation on cell viability, absorbance was expressed as numerical values, which were then statistically analyzed.

At each time interval, the cell viability percentage was calculated by dividing the numerical number of MTT optical density (OD) of each irradiation group by the OD of the control group and then multiplying by 100, according to the following equation [26]:

$$\text{Cell viability}\,{ \%}=(\text{OD irradiated group}/\text{ OD control group})\text{ X}100$$

The cell inhibition rate was calculated using this equation.

$$\text{Cell inhibitory rate }= 100\mathrm{\% }-\text{ Cell viability}\ { \%}$$

#### Serological evaluation of HBVsvps

At the end of each incubation time interval (zero, 24, 48, and 96 h) post-laser irradiation, we quantitatively measured the HBsAg by ELISA at the hepatoma cell line HepG2.2.15 supernatant for various groups (A, B, C, D, and E), following the manufacturer’s instructions (Cam Tech Medical) [24]. The color formed on the microplate reader was examined at a wavelength of 450 nm. Following that, the OD readings were averaged. where cut-Off = negative control (Mean OD) + 0.06; therefore, the absorbance (OD) was mathematically stated using the following equation: Sample /cut-off = numerical number (index).

The absorbance was expressed in numerical values by index unit, which was subjected to statistical analysis to determine the effect of LLLT on HBVsvp production from HepG2.2.15.

### Electron microscope morphometric analysis

After 96 h of laser irradiation, HepG2.2.15 and HepG2 cells were harvested, washed twice in PBS, centrifuged, fixed for 12 h in 2.5% glutaraldehyde, for 1 h in 1% osmium tetroxide, dehydrated in a graded series of acetone, and embedded in Epon 812. Then, 70–80 nm sections were cut, stained with uranyl acetate and lead citrate, and examined using a transmission electron microscope (TEM) (EM 208S, Philips, USA) [27, 28].

### Statistics

All tests were conducted in hexagons (*n* = 6) for statistical purposes and the results were averaged. The experiments were performed in triplicate (*n* = 3) with statistical analysis. The normal assumption was checked using the Shapiro–Wilk test on data that were statistically reported in terms of mean ± and standard deviation (± SD). The interaction between groups and time was examined using two-way analysis of variance (ANOVA) with repeated measures.

## Results

### Cell morphology

**Fig. 2:**
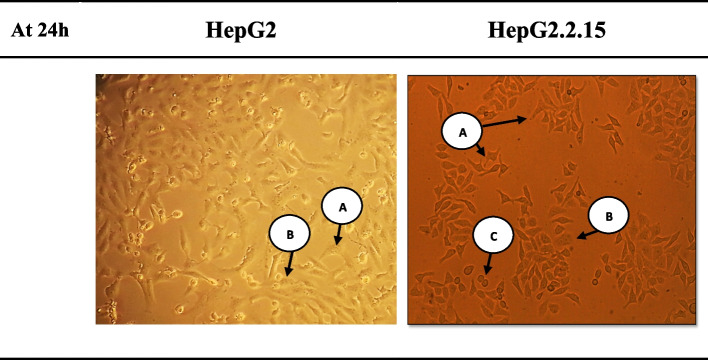
Morphology of HepG2 and HepG2.2.15 cell lines examined using phase-contrast light inverted microscope, after 24 h. culturing before laser irradiation. **A** Flattened, polygonal cells, arranged in monolayer, **B** cells with two nucleoli, and **C** HepG2.2.15 cells with a circular morphology. The magnification power is × 200

**Fig. 3:**
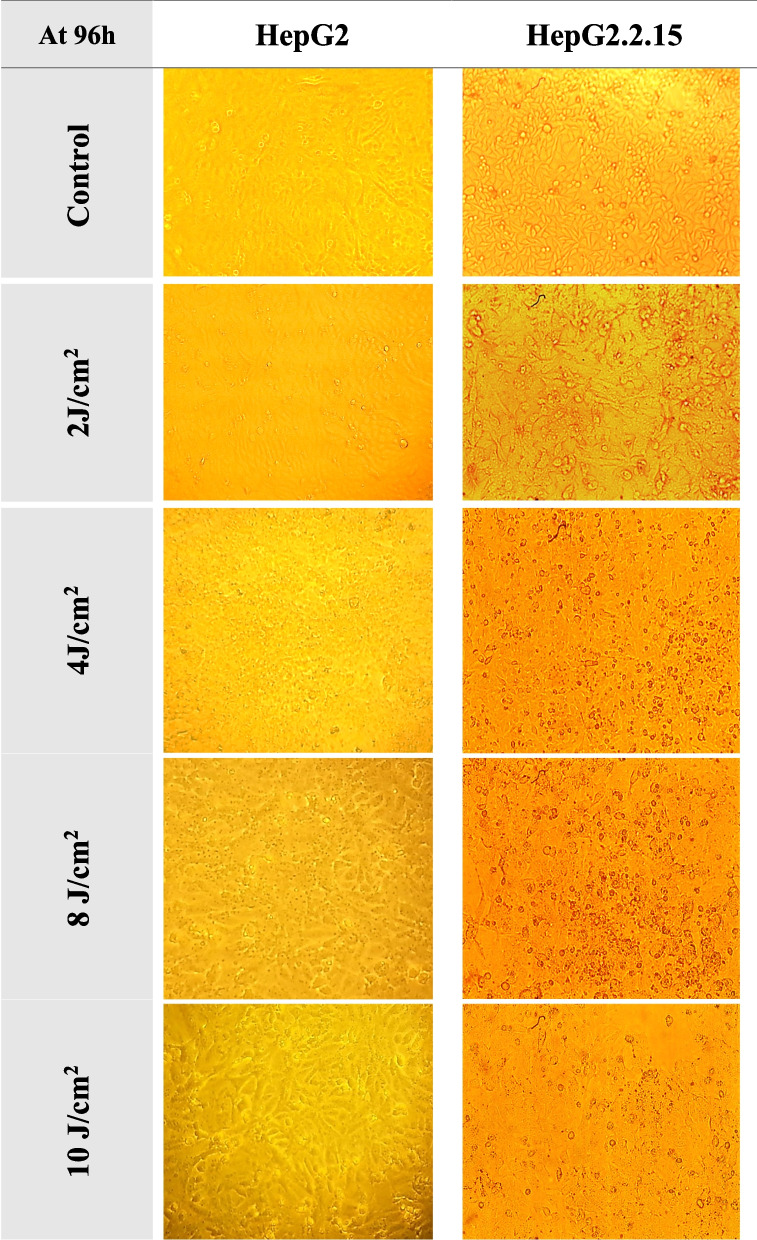
HepG2 and HepG2.2.15 cell morphology examined by phase contrast light inverted microscope at 96 h. × 100 post-laser irradiation, no morphological alterations were observed in both cell lines (HepG2 and HepG2.2.15); however, there was a decrease in cells condensation in irradiated groups of 4, 8, and 10 J/cm.^2^

Morphological examination of hepatoma cell lines HepG2.2.15 and HepG2 showed flattened, polygonal cells arranged in a monolayer, with some cells having two nucleoli (Fig. 2). The hepatoma cell line HepG2.2.15 was grown in multiple adherent layers, with circular cells visible after 96 h of division. The hepatoma cell lines, HepG2 and HepG2.2.15, produced a full sheet after 96 h. Cells were incubated for different time intervals (zero, 24, 48, and 96 h). After laser irradiation, no morphological changes were recorded in either cell line (HepG2 and HepG2.2.15), and only a decrease in cell condensation was recorded in the irradiated groups at 4, 8, and 10 J/cm^2^ (Fig. 3).

### Cell viability

#### Hepatoma cell line HepG2 viability evaluation post-laser irradiation (dose- and time-dependent)

**Fig. 4:**
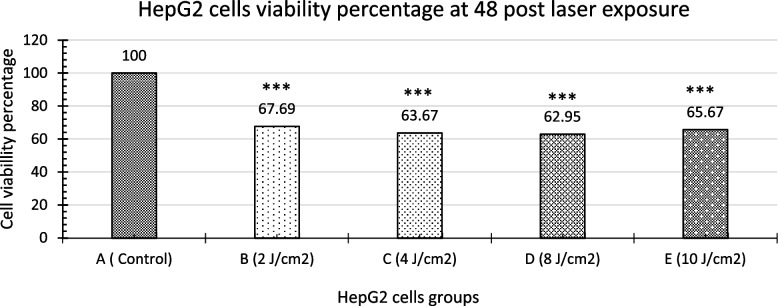
The survival rate of HepG2 cells and comparisons of the cell viability percentages between non-irradiated (**A**) and irradiated (**B**, **C**, **D**, and **E**) groups after 48 h incubation post-laser irradiation using MTT Assay. On the bar columns, significant differences between control (**A**) and their respective experimental groups are donated by (***) = *P* < 0.001

**Fig. 5:**
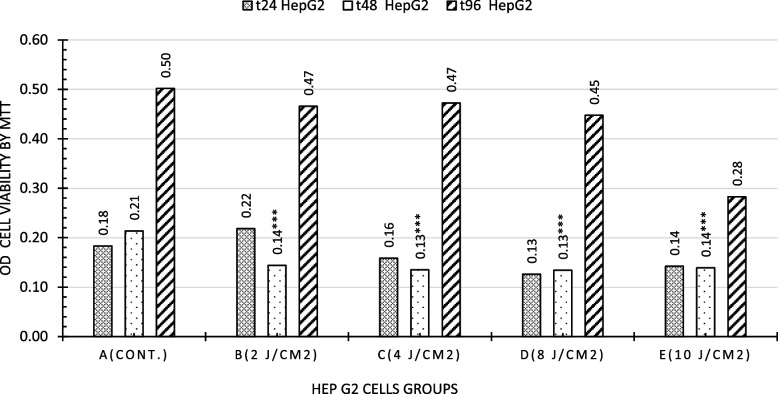
The viability of HepG2 cells in nonirradiated (**A**) and irradiated (**B**, **C**, **D**, and **E**) groups after 24 h, 48 h, and 96 h incubation post-laser irradiation using MTT Assay. Significant differences between the control (**A**) and their respective experimental groups are represented on the bar columns as (***) = *P* < 0.001

Evaluation of HepG2 cell line proliferation using the MTT assay after 48 h of incubation after laser irradiation showed a significant decrease (*P* < 0.001) in the viability of the cells for all irradiated groups from 2 J/cm^2^ to 10 J/cm^2^ in comparison to group A (control) (Fig. 4). On the other hand, after 24 h and 96 h of incubation after laser irradiation, the HepG2 changes in the cell viability results for irradiated groups (B–E) showed a non-significant decrease (*P* > 0.05) in the viability of the cells for all irradiated groups from 2 J/cm^2^ to 10 J/cm^2^ in comparison to group A (control) (Fig. 5).

#### Hepatoma cell line HepG2.2.15 viability evaluation post-laser irradiation (dose- and time-dependent)

**Fig. 6:**
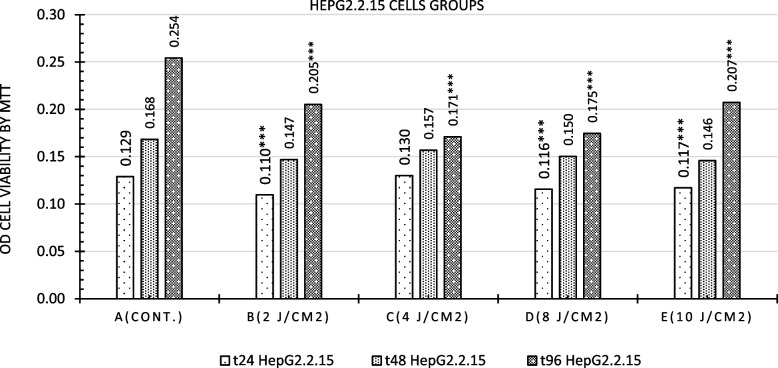
The comparisons of the HepG2.2.15 cells viability between non-irradiated (**A**) and irradiated (**B**, **C**, **D**, and **E**) groups at 24 h, 48 h, and 96 h incubation post-laser irradiation using MTT Assay. Significant differences between control (**A**) and their respective experimental groups are represented on the bar columns as (***) = *P* < 0.001

**Fig. 7:**
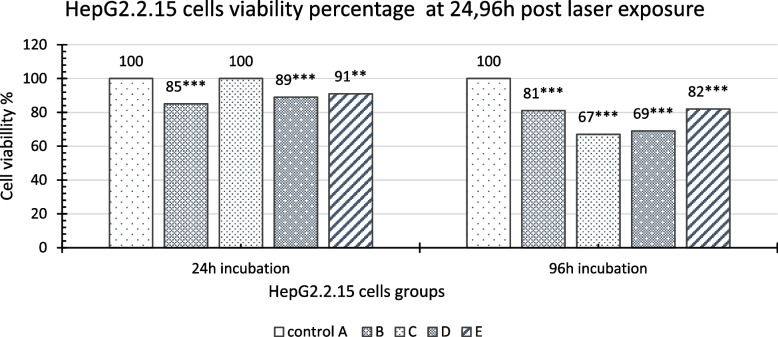
The comparisons of the cell viability percentage of HepG2.2.15 cells between non-irradiated (**A**) and irradiated (**B**, **C**, **D**, and **E**) groups at 24- and 96-h incubation post-laser irradiation by MTT Assay. Significant differences between control (**A**) and their respective experimental groups are represented on the bar columns as (**) = *P* ≤ 0.01, (***) = *P* < 0.001

The changes in cell proliferation of the hepatoma cell line HepG2.2.15 irradiated groups (B, D, and E) after 24 h of incubation after laser irradiation showed a significant decrease (*P* < 0.001) compared to the control group (A), while after 96 h of incubation post-laser irradiation, all irradiated groups (B–E) showed a significant decrease (*P* < 0.001) compared to the control group (A). In contrast, 48 h of incubation after laser irradiation for all irradiated groups (B–E) showed a non-significant decrease (*P* > 0.05) in the viability of the cells when compared to the control group (A) (Figs. 6 and 7).

### HBVsvp measurements post-laser irradiation: dose- and time-dependent

**Fig. 8:**
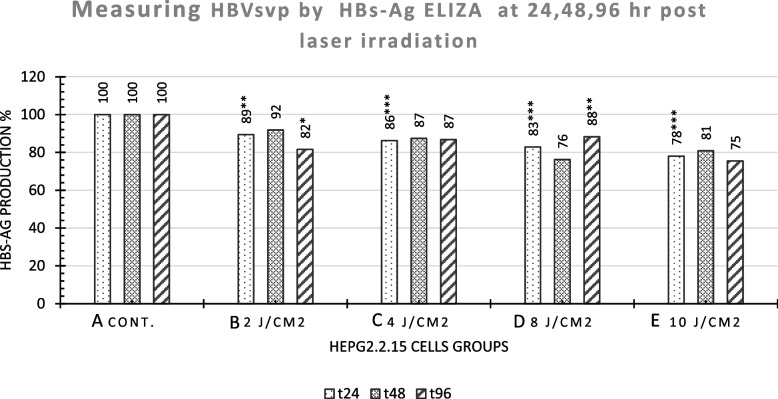
HBVsvps concentration produced in the HepG2.2.15 cells supernatant. Percentage comparisons between the concentration in the supernatant of the non-irradiated group (**A**) and that in irradiated groups (**B**, **C**, **D**, **E**) at 24, 48, and 96 h incubation post-laser irradiation by HBsAg ELISA Assay. *P* ≤ 0.01 (**), *P* ≤ 0.001 (***)

After 24 h of incubation following laser irradiation, the concentration of HBVsvp in the cell culture supernatant produced by all irradiated groups (B, C, D, E) was significantly lower (*P* < 0.001) than that in group A (control). After 48 h and 96 h of incubation post-laser irradiation, the concentration of HBVsvp in the cell culture supernatant produced by all irradiated groups (B, C, D, E) compared to group A (control group) showed a non-significant decrease in HBVsvp level, except for both irradiated groups B and D, which showed a significant decrease (*P* < 0.05) and (*P* < 0.01) compared to the control group A at 96 h (Fig. 8).

### Electron microscope morphometric analysis

**Fig. 9:**
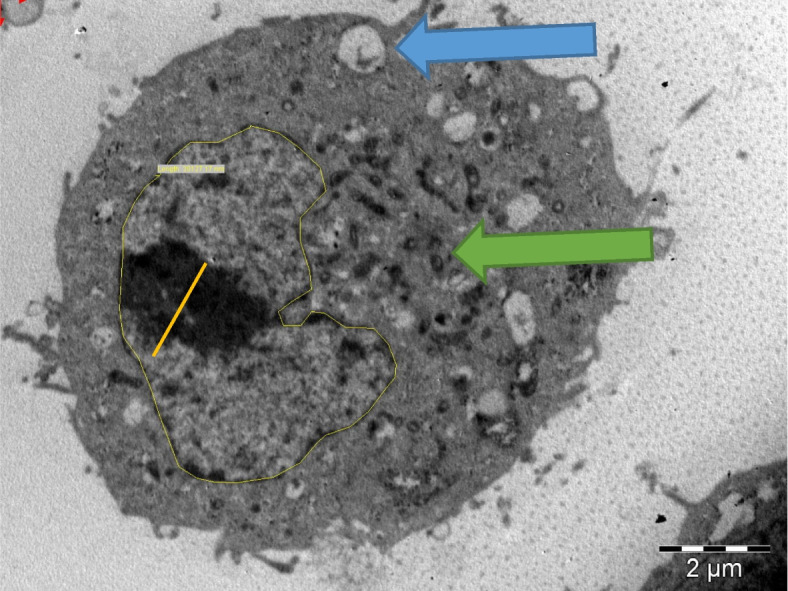
HepG2 cells of the control group (non-irradiated cells) showed a fairly regular cell membrane. A moderate number of small mitochondria, (green arrow). Oval shape euchromatic nucleus with irregular nuclear membrane, (yellow line), nucleus surface area length was 2012717 μm.^2^. A large peripheral irregular nucleolus, nucleolus diameter (1.6 μm). Autophagy vacuoles, (blue arrow). × 5600

**Fig. 10:**
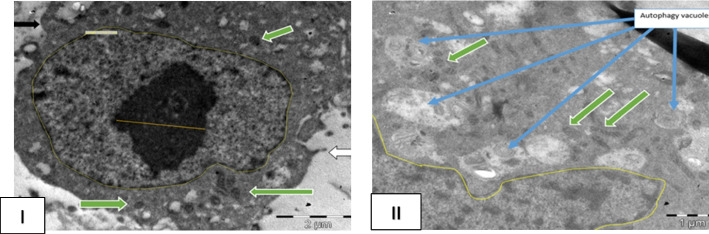
HepG2 cells of group E (10 J/cm^2^), showed **I** reduced number of mitochondria with preserved cristea, (green arrows). Euchromatic nucleus with fairly regular nuclear membrane, (yellow line), Nucleus surface area length was 1710.71 μm.^2^. A large peripheral irregular nucleolus, (yellow bar), nucleolus diameter (2.5 μm), 8900 X. **II** Mitochondria with preserved cristea, (green arrows). Many autophagy vacuoles (blue arrows). × 11,000

**Fig. 11:**
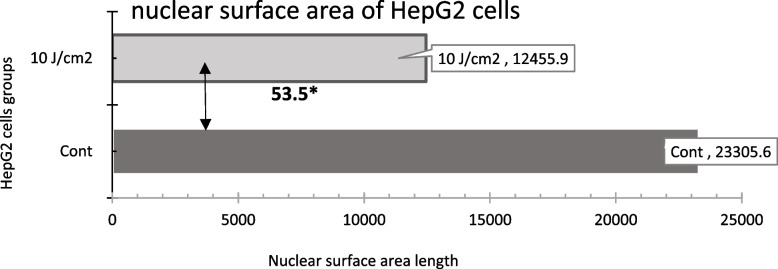
The comparisons of the nuclear surface area of HepG2 cells between non-irradiated group (**A**) and irradiated groups (**E**) at 96 h incubation post-laser irradiation by TEM Assay. Significant differences between control (**A**) and their respective experimental groups are represented on the bar columns as *P* ≤ 0.05 (*)

According to transmission electron microscopy examinations after 96-h post-laser irradiation, HepG2 cells in group A (control) had a fairly regular cell membrane, an euchromatic nucleus, an irregular nuclear membrane, an oval nucleolus, a large diameter (1.6 μm), a tiny mitochondrion, and few fatty droplets (Fig. 9). In comparison with group A (control), the electron micrograph of HepG2 cells in group E (10 J/cm^2^) revealed an increase in the prevalence of autophagy vacuoles, as well as less regularity in the nuclear membrane in the irradiated group E (Fig. 10). Furthermore, the nuclear surface area comparison revealed a significant decrease (*P* ≤ 0.05) in group E (10 J/cm^2^) of approximately 53% compared to non-irradiated cells (Fig. 11).

**Fig. 12:**
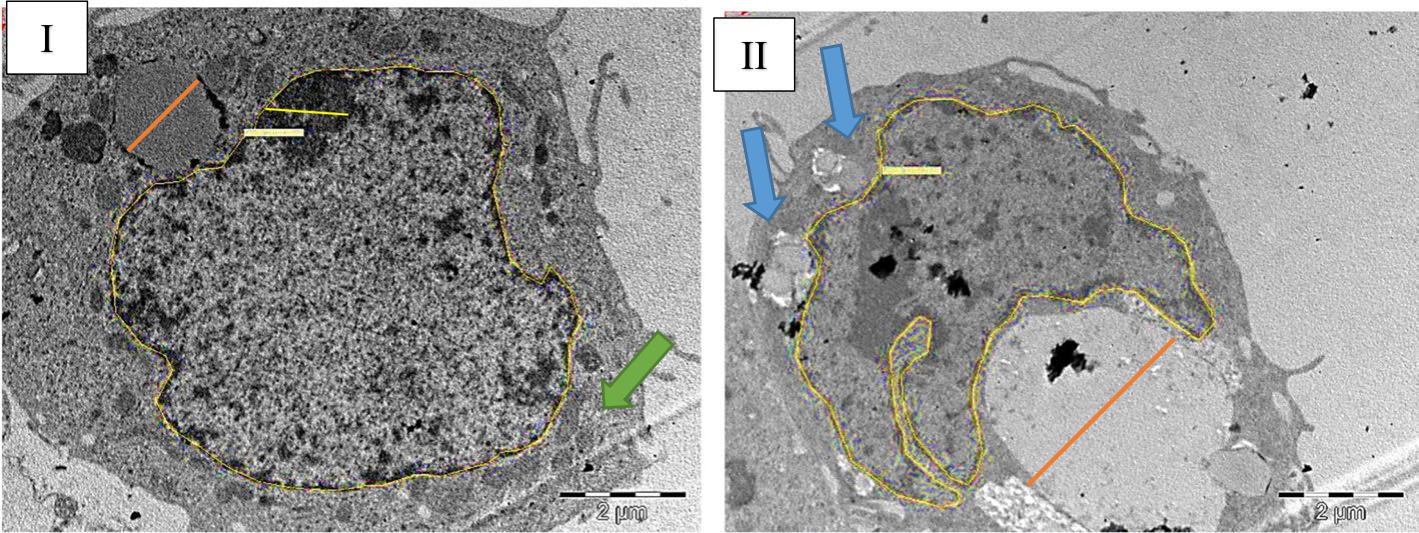
HepG2.2.15 cells of the control group (non-irradiated cells) showed **I** fairly regular cell membrane. Euchromatic nucleus with irregular nuclear membrane, (yellow line), and peripheral nucleolus, (yellow bars), the diameter of the nucleolus (1.5 μm), nucleus surface area length was (24,196.90 μm^2^). Fat droplet, (orange par), the diameter was (1.7 μm). Different size mitochondria, with preserved cristea, (green arrows). × 5600. **II** Fairly regular cell membrane. Euchromatic nucleus with irregular nuclear membrane, (yellow arrow). Nucleus surface area was (30084.75 μm^2^). Fat droplets, (orange arrows), the diameter was (3 μm). Autophagosomes (blue arrow). × 5600

**Fig. 13:**
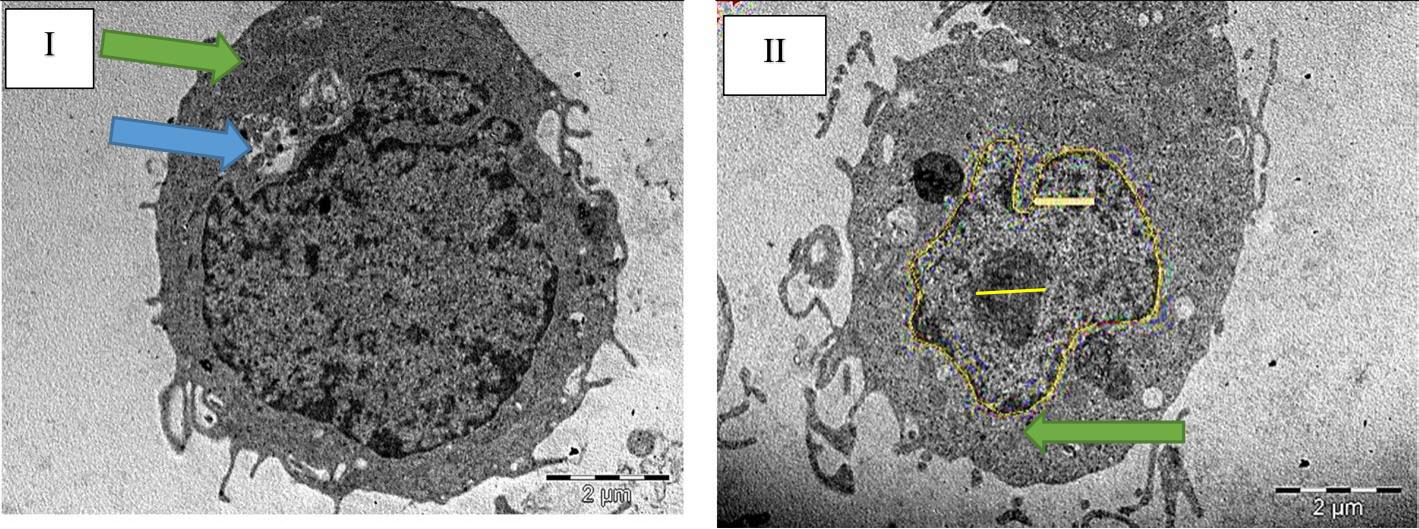
HepG2.2.15 cells of irradiated group E (10 J/cm^2^). **I** Euchromatic nucleus with irregular nuclear membrane (yellow line). Reduced number of mitochondria, (green arrows). Autophagy vacuoles (blue arrow). × 7100. **II** Euchromatic nucleus with markedly irregular nuclear membrane (yellow line), nucleus surface area length was (1518537 μm^2^). Large par central nucleolus, the diameter was (1.2 μm). Mitochondria, with preserved cristea, (green arrows). × 7100

**Fig. 14:**
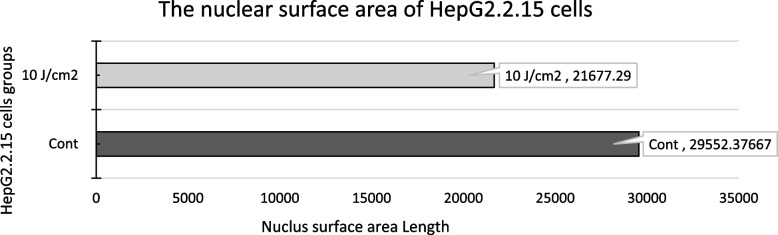
The comparisons of the nuclear surface area of HepG2.2.15 cells between non-irradiated (**A**) and irradiated (**E**) groups at 96-h incubation post-laser irradiation, as measured by the TEM Assay

After 96 h of laser irradiation, ultrastructure analysis of control hepatoma HepG2.2.15 cells in group A revealed a regular cell membrane and variable-sized mitochondria with intact cristea. Euchromatic nucleus with irregular nuclear membrane and large nucleocytoplasmic ratio (N/C ratio). The nucleolus was peripheral of 1.5 μm diameter (Fig. 12I) and peripheral, irregular, and elongated in shape (Fig. 12II). Large fat droplets were observed (Fig. 12II). Autophagosomes were also present (Fig. 12II). Electron micrographs of the hepatoma HepG2.2.15 cell line from irradiation group E (10 J/cm^2^) showed an euchromatic nucleus with an irregular nuclear membrane. Autophagic vacuoles were also observed (Fig. 13I). In addition, a comparison of the nuclear surface area between group A (control) and HpG2.2.15 cells in group E (10 J/cm^2^) revealed a non-significant decrease (*P* ≥ 0.05) of approximately 26.6% smaller than that in the control (Fig. 14).

## Discussion

Several studies have shown that LLL therapy at various wavelengths may be beneficial for viral infection. For example, green light lasers enhance tissue oxygenation, while blue light lasers are utilized in photobiomodulation therapy (PBMT) [29]. Lugongolo et al. in 2020 showed that PBMT enhanced apoptosis in human immunodeficiency virus (HIV-1)-infected cells but had no inhibitory effects on HIV-1 uninfected cells [30]. The goal of this study was to evaluate the in vitro growth behavior and hepatitis B subviral particle secretion in a biomodulated human hepatoma HepG2.2.15 cell line using (650 nm) low-level laser in doses effects. Our findings showed changes in cell viability and ultrastructure in both hepatoma cell line HepG2.215 and HepG2 cells, in addition to a reduction in HBVsvp production in response to LLLT irradiation, in a dosage- and time-dependent manner.

The efficacy of LLLT is regulated by numerous factors, such as wavelength, power output, energy density, and duration of radiation. However, considering the diversity of laser parameters, an accurate effective dose has not yet been established [31]. In our study, we utilized dose fluencies falling between 2 and 10 J/cm^2^ that has an inhibitory effect on cell lines and in the same line with the analysis of the current literature found that doses between 0.001 to 10 J/cm^2^ provide the ideal therapeutic window for photobiomodulation [32].

The human hepatoma cell line HepG2.2.15, containing an integrated HBV genome, was used as an expression system to produce HBVsvp [3]. In the present study, selection of the human hepatoma HepG2.2.15 cells transfected with HBV surface region as it promotes a stable HBV replication, production of HBVsvps as well as a very reach organ by cytochrome C oxidase enzyme containing mitochondria which is the specific chromophore of red laser light absorption [24, 33]. In vitro cell culture models, such as human hepatoma HepG2 cells, also constitute potent cell models for HBV and have been used in an increasing number of studies [24, 29]. The concentration of HBVsvps was measured in the cell culture supernatant of each laser-treated human hepatoma HepG2.2.15 cells groups at 2 J/cm^2^, 4 J/cm^2^, 8 J/cm^2^, 10 J/cm^2^ and the non-irradiated group (control), and the results of HBVsvp levels were compared in a dose- and time-dependent manner after laser irradiation in triplicate.

Before laser irradiation, morphological analysis of both human hepatoma HepG2 and human hepatoma HepG2.2.15 cell lines demonstrated no morphological changes with growth as flattened polygonal cells arranged in a monolayer, with certain cells having two nucleoli. Furthermore, human hepatoma HepG2.2.15 cells were grown in multiple adherent layers, and at late stages of growth, a circular form was observed (after 96 h) (Figs. 2 and 3). These findings are in line with those of Wang et al., who described the morphological characteristics of hepatocellular carcinoma cells (HepG2 and HepG2.2.15) [24].

Following laser irradiation (2–10 J/cm^2^), we examined the morphology of human hepatoma HepG2 cells and HepG2.2.15. Our findings showed that there were no morphological changes induced in all irradiated groups compared to the non-irradiated control group over incubation time intervals ranging from 24 to 96 h post-laser irradiation. This result may be due to the use of a one-shot low-output laser power (35 mW) that does not cause heating; even higher doses of 8 J/cm^2^ and 10 J/cm^2^ were obtained by increasing the exposure time, not by increasing the laser output power, which is in agreement with previous studies that applied variable laser power, 30 mW [34] and 100 mW LED, both of which showed a reduction in oral mucositis**.** In our study, a relative decrease in human hepatoma HepG2 cell condensation was observed in both irradiated groups, D (8 J/cm^2^) and E (10 J/cm^2^). In human hepatoma HepG2.2.15, all irradiation groups showed a relative decrease in cell condensation, with a relative increase in circular cells in the irradiated C (4 J/cm^2^), D (8 J/cm^2^), and E (10 J/cm^2^) groups (Fig. 3). This is in agreement with [30].

After laser irradiation (2–10 J/cm^2^), the cell proliferation rate evaluated by MTT assay in a dose- and time-dependent manner between the irradiated and control groups showed that after 24 h of incubation (short-term effect), human hepatoma HepG2.2.15 irradiated at 2 J/cm^2^, 8 J/cm^2^ and 10 J/cm^2^ showed a significant decrease in viability compared with the control group (*P* < 0.001) with a maximum inhibitory dose of 2 J/cm^2^ (Figs. 6 and 7). This effect, which, according to some previous studies, can be interpreted as a red laser promoting short-term activation of the respiratory chain, resulting in alterations in the redox state of both the mitochondria and cytoplasm [35]. Our results are inconsistent with those of Guimaraes et al., who found that applying 2 J/cm^2^ from a red diode laser with a wavelength of 660 nm and power of 100 mW reduced oral mucositis [36]. Our findings contradict those of other studies, which found no significant differences in cell proliferation of TZM-bl cells after 24-h incubation following red diode laser irradiation (660 nm), (100 mW) CW mode, with fluences of 2–10 J/cm^2^ [26], and in cell proliferation of human gingival fibroblasts after irradiating cells with increasing fluences of 0.5 J/cm^2^, 1.5 J/cm^2^, 3.5 J/cm^2^, and 7 J/cm^2^ [37]. This variation can be attributed to the nature of the cell line employed.

The lack of a direct correlation between dosage duplication and the effect produced is evident from our study’s cell proliferation examination where after 24-h incubation post-laser irradiation (short term effects) (Fig. 7), the cell proliferation of human hepatoma HepG2.2.15 cells irradiated with 2 J/cm^2^ dose was decreased by 15%, while 8 J/cm^2^ caused a decrease of 11%, finally, 10 J/cm^2^ induced decrease in cell viability by 9%. These fluctuations can be explained by the dose-dependence of the effect. This finding was reported in a previous study that used head and neck squamous cell carcinoma and applied single irradiation delivering 1 J/cm^2^ or 2 J/cm^2^ and observed that 1 J/cm^2^ promoted an increase in cell proliferation, while no effect was observed with 2 J/cm^2^ [38].

Our HBVsvps concentration results showed that the short-term (after 24 h incubation) effect of LLLT at all irradiation doses 2 J/cm^2^, 4 J/cm^2^, 8 J/cm^2^, and 10 J/cm^2)^ on HBVsvps production was significantly decreased when compared to a non-irradiated control group, with a maximum inhibitory effect at 10 J/cm^2^ dose (*P* < 0.001) (Fig. 8). This agrees with a previous study in which the viral load was reduced by LLLT [39].

Regarding the moderate effect (after 48-h incubation) post-laser irradiation, the cell viability analysis of HepG2 cells showed a significant decrease with irradiated doses of 2 J/cm^2^, 4 J/cm^2^, 8 J/cm^2^, and 10 J/cm^2^ when compared to the control group (*P* < 0.001) (Fig. 4), and the maximum effect was recorded for both doses 4 J/cm^2^ and 8 J/cm^2^, which agrees with data from a previous study reporting reduced incidence of oral mucositis to 53% when compared to the untreated group (70–90%) by using 4 J/cm^2^ red laser therapy [40] and with the—in vitro—study of [41] they used LLLT doses (5 J/cm^2^ and 10 J/cm^2^) at 685 nm, 50 mW, on cancer (HeLa) cells and the cell viability by MTT was decreased 48 h after laser irradiation.

In contrast, after 48 h, when human hepatoma HepG2.2.15 cells were irradiated with doses ranging from 2 J/cm^2^ to 10 J/cm^2^, no significant variations in cellular viability or HBVsvps production were observed when compared to the control group (Figs. 6 and 8). This could be explained by the fact that different cells respond differently to light therapy depending on the nature of the cell line used. Our results are in agreement with those in [42]. They recorded that a 4.8 J/cm^2^ red laser (635 nm) did not stimulate HepG2 cell proliferation.

In this study, human hepatoma HepG2 and HepG2.2.15 cells were viable after 96 h of laser irradiation (late cellular response) and showed an insignificant decrease in HepG2 cells at all irradiation doses (2, 4, 8, and 10 J/cm^2^) (Fig. 5). The viability of human hepatoma HepG2.2.15 cells with irradiated doses (2 J/cm^2^, 4 J/cm^2^, 8 J/cm^2^, and 10 J/cm^2^) significantly decreased when compared to the control group (*P* < 0.001) (Figs. 6 and 7), and the maximum effect was recorded for the 4 J/cm^2^ dose, which is compatible with our morphological observation by inverted microscopy regarding the decrease in human hepatoma HepG2.2.15 cell condensation after 96 h (Fig. 3). While the inhibitory effect on HBVsvps concentration production compared to non-irradiated cells (control), all irradiated dose of 2 J/cm^2^, 4 J/cm^2^, 8 J/cm^2^, and 10 J/cm^2^ showed a decrease in HBVsvps production, with 10 J/cm^2^ being the maximum inhibitory dose (Fig. 8). Our late response findings agree with a study by Barasch and his team on HeNe 632.8 nm, applied energy density (4 J/cm^2^), single application, on leukemic human cells (HL60), cell viability was decreased at day 3, 4, and 6, analysis every 24 h for 6 days [43], but in contrast with Coombe et al. who found no significant early (24 h post-laser irradiation) or late (after10 days post-laser irradiation) effects of laser irradiation on protein expression and alkaline phosphatase activity [44], which could be attributed to utilizing different cell seeding density (20,000 cells per well) and LLLI irradiated fluencies of 0.27 J/cm^2^, 1 cm^2^, and 4 J/cm^2^ daily, while in our study, the seeding density of human hepatoma HepG2 and HepG2.2.15 was 10,000 cells irradiated only once.

The current study demonstrated that even when administered the same dose of LLLT, each cell line responded differently to it, where the effect of irradiation with the highest (10 J/cm^2^) dose on HepG2 cells after 96-h (long effect) recorded a maximum non-significant reduction of 45% compared to the control, whereas in human hepatoma HepG2.2.15 cells at 10 J/cm^2^, a minimum significant inhibitory effect of 18% was observed compared to the control. This is incompatible with the results of a previous study that used a diode laser at 636 nm and 5, 10, and 20 J/cm^2^ (exposure times = 534, 1068, and 2136 s). single application on lung cancer cells (A549), 20,000 cells were analyzed after 24 h, 48 h, and 72 h. Increases in all doses at 72 h [45] may be due to the number of cells 20,000 cells, while in our study 10,000, in addition, the exposure time for 10 J/cm^2^ was too long (1068 s). In our study, the laser power was 35 mW, and the exposure time was 36 s.

The results of HBVsvps production were partially compatible with the results of human hepatoma HepG2.2.15 cell viability by MTT, whereas the early effect of irradiation laser dose showed a significant decrease in both (human hepatoma HepG2.2.15 cell viability and HBVsvps production) except that group C (4 J/cm^2^) showed a non-significant decrease in viability (Figs. 7 and 8). In addition, the late effect (after 96 h), all irradiated human hepatoma HepG2.2.15 cell groups (B, C, D, and E) showed a significant decrease in cell viability, while a significant decrease in HBVsvps expression was recorded only in the supernatant of both irradiated groups B (2 J/cm^2^) and E (8 J/cm^2^) (Figs. 7 and 8). As a result, we believe that the mechanism of action that affects cell viability after laser irradiation is distinct from that affecting viral expression, and further research is needed. This agrees with [6], who recorded a bifacial effect of LLLT in chronic viral patients.

Our results showed that the maximum decrease in HBVsvps was recorded at 96 h in group E (10 J/cm^2^). Therefore, we studied ultrastructural changes in human hepatoma HepG2.2.15 cells and Hepg2 cells irradiated in group E (10 J/cm^2^).

Ultrastructural changes were compared between the irradiated HepG2 cells group E (10 J/cm^2^) and a non-irradiated control group A at 96 h. showed an increase in autophagy vacuoles, a decrease in the regularity of the nuclear membranes (Figs. 9 and 10), and a significant decrease (*P* < 0.05) in the nuclear surface area in group E (10 J/cm^2^) by approximately 53% compared to the control group (Fig. 11). This finding is consistent with that of Lynnyk et al. (2018), who investigated the mechanisms involved in the death process of the human hepatic cell line Huh7 under laser irradiation. The Lynnyk group decoupled distinct cell death pathways targeted by laser irradiation at different powers. Their data demonstrated that high-dose laser irradiation resulted in the highest levels of total reactive oxygen species production, leading to cyclophilin D-related necrosis via mitochondrial permeability transition. In contrast, low-dose laser irradiation results in the nuclear accumulation of superoxide and apoptosis execution [46, 47].

Electromicrographs of human hepatoma HepG2.2.15 cells from the irradiated group E (10 J/cm^2^) in comparison to the non-irradiated group A showed euchromatic nuclei with irregular nuclear membranes, but less than those in the control group A. Autophagy vacuoles were large, and some vacuoles were up to 1 μm in diameter (Figs. 12 and 13). Their nuclear surface area was not significantly decreased compared to that in the control group (*P* ˃ 0.05) (Fig. 14). Our findings agree with those of previous studies that evaluated the ultrastructural features of HepG2 cells, and the hepatocyte cell model induced viral replication and propagation of HBV for a long time 20 days and were compared with non-infected cells [48, 49].

An explanation for our morphometric findings in human hepatoma HepG2 and HepG2.2.15 from ultrastructure electrophotography evaluation leads us to believe that the decrease in cell condensation and viability is related to decreased proliferation ability, as evidenced by the decrease in the nuclear surface area induced by LLLI compared with the non-irradiated control group. This reduction in nuclear surface area might denote the arrested proliferating state of these cells because proliferating cells commonly show prominence in their nuclei, for example, malignant cells of human liver cell carcinoma [50]. Our findings agree with those of a previous study, suggesting that LLLI inhibits the proliferation of human hepatoma HepG2.2.15 cells by regulating cell cycle gene expression and inducing G1 phase arrest [24]. Moreover, Lynnyk et al. (2018) has been shown that red laser light may initiate apoptosis via the induction of reactive oxygen species-mediated mitochondrial permeability transition [27–51] they have also been reported that red light-induced cell damage is mainly caused by the production of reactive oxygen species (ROS) in the mitochondria.

## Conclusion

The results of the study indicate that LLLT administered at varying fluences (2–10 J/cm^2^) led to a significant reduction in viability, proliferation, and HBVsvp production in the human hepatoma cell lines HepG2 and HepG2.2.15. Furthermore, this effect was dose, cell type, and time-dependent. These findings indicate a significant implication of the photobiomodulation effects caused by LLLI in the clinical context, as they suggest that LLLT may serve as a promising therapeutic modality (in combination with current anti-HBV therapy) in patients with HCC or HBV infections, as it may help mitigate the HCC progression by reducing the proliferation of malignant cells and limiting the production of HBVsvp, further investigation needed for clinical applications. In addition, the varied response of hepatoma cells (HepG2.2.15 cells were more *sensitive* than HepG2 cells) to LLLT highlights the need for developing standardized LLLT protocols, particularly in clinical settings to patients with HCC, and HBV infection due to HCC’s heterogeneous nature, accounting for various LLLT parameters (wavelengths, exposure, and incubation time post-laser irradiation, intensities …etc.) that influence personalized treatment plans for optimal outcomes which is highly advisable. Furthermore, the fact that HepG2.2.15 cells were more responsive to LLLT than HepG2 cells (the varied response of hepatoma cells) emphasizes the importance of developing standardized LLLT protocols, especially in clinical settings where patients with HCC and HBV infection are treated. Due to the heterogeneous nature of HCC, various LLLT parameters (wavelengths, exposure, and incubation time post-laser irradiation, intensities …etc.) need to be considered when developing personalized treatment plans for optimal outcomes.

Our study found that high doses induce ultrastructural changes in mitochondria and nuclear membranes, which could be possible mechanisms involved in the death process of HepG2 and HepG2.2.15 human hepatoma cell lines, including reactive oxygen species and necrosis, in comparison to low dose induction of apoptosis. This study suggests that laser therapy could potentially be used to target mitochondrial and nuclear activation in HCC patients. However, further research is needed to determine the photobiological changes induced in HCC cell line by LLLT doses, the optimal dosing, and treatment protocols to achieve the desired outcome. To improve the accuracy and reliability of assessing the effect of LLL irradiation on HepG2 and HepG2.2.15 human hepatoma cell lines, including proliferation and apoptosis, certain recommendations should be considered. Primarily, immunohistochemistry can be used to investigate marker expression in distinct phases of the cell cycle, particularly the S phase, resulting in a more accurate estimate of proliferation and apoptosis rates. Additionally, imaging techniques such as confocal or fluorescence microscopy, in conjunction with EM, can assist in visualizing the markers’ localization and distribution within the hepatoma cell line. These suggestions aim to provide a more comprehensive understanding of the cell response to LLLI, resulting in improved analytical precision and dependability. Collaborating with experts from fields like pathology, oncology, and imaging can offer insights into the photobiomodulation mechanisms caused by LLLI. This interdisciplinary approach can enhance our understanding of LLLI's impact on hepatoma cells and aid in developing effective therapeutic strategies.

## Data Availability

The datasets generated or analyzed during this study are included in this article and the corresponding author will support any supplementary information files on reasonable request.

## Abbreviations

HBV: Hepatitis B virus
HCC: Hepatocellular carcinoma
LLLT: Low-level laser therapy
HBVsvp: Hepatitis B virus subviral particles
NAs: Nucleoside/nucleotide analogs
PBM: Photobiomodulation
NIR: Near-infrared
